# Post-COVID Adolescent Public Health

**DOI:** 10.34763/jmotherandchild.20212503SI.pov.2021_25_03SI

**Published:** 2022-02-09

**Authors:** Joanna Mazur

**Affiliations:** 1 Department of Humanization in Medicine and Sexology Institute of Health Sciences Collegium Medicum, University of Zielona Góra, Zielona Góra, Poland

## Post-COVID adolescent public health

This special issue of the *Journal of Mother and Child* has been focused on current directions in the prevention of health problems in children and adolescents. It is worth evaluating to what extent the topics taken up by the authors fit into the contemporary understanding of the tasks in public health. The purpose of my brief commentary is also to draw attention to the importance of research in public health and to the suggested directions for the development of health education in its basic version and in the expanded school curriculum [1].

In the history of public health over the past few decades, a series of consecutive phases can be highlighted. Initially, the focus was on combating infectious diseases. Then attention was paid to the development of appropriate lifestyles as a method of prevention of civilization diseases, which also resulted in the golden age of health promotion. In subsequent phases, more attention was paid to strengthening health systems and promoting evidence-based interventions. However, the reintegration of infectious diseases into priority areas has been evident for many years, and the COVID-19 pandemic accelerated this process. In contemporary public health, new concepts like One Health and Planetary Health are increasingly talked about. *One Health* has been defined by the WHO as ‘an approach to designing and implementing programmes, policies, legislation and research in which multiple sectors communicate and work together to achieve better public health outcomes’ [2]. *Planetary health* is a new term being used for environmental health. It includes such topics as vector-borne diseases, water and food security, population growth and migration, and extreme climatic events. Both these ideas point to growing environmental risks; adverse trends include, but are not limited to, biodiversity loss, climate change, particulate air pollution, ocean acidification, and deforestation. Experts draw attention to the existing co-occurrence of two crises that will certainly affect the health of populations [3]. On the one hand, there is the climate crisis, and on the other a pandemic, and these two interrelated phenomena are exacerbating social inequalities in health.

Young people are showing great interest in the health of the planet, as exemplified by youth activists such as Greta Thunberg. Deepening knowledge on contemporary environmental threats to population health can be included as an extension of education for health literacy, at different levels of sophistication. Experts agree on the need to integrate One Health and Planetary Health concepts into academic and postgraduate education. Related content can also enter school education in the older grades, more broadly than ecological education, which is provided also in the core curriculum at earlier stages of learning. These are the problems that future generations will be most affected by; they will bear the consequences of today's negligence. As I mentioned earlier, young people are showing a tremendous willingness to act. The aspect of contemporary threats was emphasized in the presented special issue in some papers (Korzycka et al., Gajda et al.) based on data collected during the COVID-19 pandemic. Furthermore, in the study by Porwit et al. attention was paid to the interest of youth in prosocial and pro-ecological activities.

Current public health emphasizes health security monitoring by identifying priority areas and methods of impact, taking into account the specifics of different countries and regions, their health systems, and existing legislation. In 2012, Essential Public Health Operations (EPHO) were developed at the WHO Regional Office for Europe. The list of ten essential operations includes three thematic sections: surveillance-related functions, public health services, and so-called enabling functions [4]. The title of this special issue highlights the middle one and namely EPHO 5 (Disease prevention, including early detection of illnes). However, is it limited to this one area? The development of research in the area of public health is mentioned in the assumptions of the EPHO system as the last supporting function. It is noteworthy that the scientific community has been quick to respond to new threats to adolescent health as a result of the COVID-19 pandemic. In addition to the immediate threat from the risk of SARS-COV2 virus infection, attention is drawn to the psychosocial health consequences of social isolation and the need to undertake remote learning.

An important source of information on adolescent health is the Health Behaviour in School-aged Children (HBSC) survey, repeated every four years, which currently covers 51 countries of the European region and Canada. The next round of these surveys, which will be conducted this school year, will make it possible to assess the effects of the COVID-19 pandemic from a national and international perspective. Thus, referring to the public health operations cited above, it is worth emphasizing the importance of international systems for collecting data on the health of children and adolescents. Research studies, including HBSC, have repeatedly been the basis for initiating prevention programs and supporting their evaluation. Recently, researchers affiliated with the HBSC network have begun to join the discussion on sustainability, valuing the possibility of their contribution. In the current round of the HBSC, there are optional packages referring to contemporary directions in public health, such as environmental health literacy and planetary health, and COVID-19 (with two related modules). Looking at these problems from a multi-country perspective will overcome the limitations of the studies published so far, which have mostly been based on data from a single country [5]. We can therefore hope that the results obtained will be translated into further intervention programmes and will help to expand the scope of school health education.

Joanna Mazur, MD, PhD, Ass. Prof.

Guest Editor

Department of Humanization in Medicine and Sexology Institute of Health Sciences Collegium Medicum, University of Zielona Góra, Zielona Góra, Poland
